# The Role of Immune Checkpoint Inhibitors in Classical Hodgkin Lymphoma

**DOI:** 10.3390/cancers10060204

**Published:** 2018-06-15

**Authors:** Nicholas Meti, Khashayar Esfahani, Nathalie A. Johnson

**Affiliations:** Department of Medicine, Jewish General Hospital, McGill University, Montreal, QC H3T 1E2, Canada; nicholas.meti@mail.mcgill.ca (N.M.); khashayar.esfahani@mail.mcgill.ca (K.E.)

**Keywords:** Hodgkin Lymphoma, immune checkpoint inhibitors, immunotherapy

## Abstract

Hodgkin Lymphoma (HL) is a unique disease entity both in its pathology and the young patient population that it primarily affects. Although cure rates are high, survivorship can be linked with significant long-term morbidity associated with both chemotherapy and radiotherapy. The most significant recent advances have been with the use of the anti-CD30-drug conjugated antibody brentuximab vedotin (BV) and inhibitors of program death 1 (PD-1). HL is genetically wired to up-regulate program death ligand 1 (PD-L1) in >95% of cases, creating a state of so-called “T cell exhaustion”, which can be reversed with immune checkpoint-inhibitor blockade. The overall and complete response rates to PD-1 inhibitors in patients with relapsed or refractory HL are 70% and 20%, respectively, with a long median duration of response of ~16 months. In fact, PD-1 inhibitors can benefit a wide spectrum of relapsed HL patients, including some who have “progressive disease” by strict response criteria. We review the biology of HL, with a focus on the immune micro-environment and mechanisms of immune evasion. We also provide the rationale supporting the use of PD-1 inhibitors in HL and highlight some of the challenges of monitoring disease response in patients treated with this immunotherapy.

## 1. Overview of Hodgkin Lymphoma

Hodgkin Lymphoma (HL) has an annual incidence of 2–3 cases per 100,000 in Europe and the USA, and epidemiological data has revealed that one in eight patients will die from the disease [1,2,3]. It has a bimodal peak, with young adults aged between 15–34 being the most affected, followed by those aged 60 and older [4]. Although the cause of HL is unknown, several risk factors have been studied. These include genetic predisposition [5], immunosuppression in the context of human immunodeficiency virus infection [6], and other viral infections such as Epstein Barr Virus [7]. Based on cellular morphology and immunohistochemistry, HL can be classified into either classical HL (cHL) or nodular lymphocyte-predominant HL (NLPHL) [8,9,10]. cHL represents approximately 90% of all HL and can be further classified into four histological subtypes known as nodular sclerosis HL, mixed cellularity HL, lymphocyte-rich HL, and lymphocyte depleted HL. Given the predominance of cHL, this review will focus on cHL and advancements in the treatment of cHL.

## 2. Biology and Diagnosis of Classical Hodgkin Lymphoma

The histological presence of mononuclear Hodgkin (H) and bi- to multi-nuclear, diagnostic Reed–Sternberg (RS) cells is pathognomonic of cHL. Given that both cell types are malignant, they are also referred to as HRS cells. They are derived from clonal germinal center B (GCB) cells, having rearranged and mutated immunoglobulin variable genes [11], However, with the exception of PAX5, typical GCB surface markers (CD19, CD79) and transcription factors (OCT-2, BOB.1 and PU.1) are down-regulated or completely absent in HRS cells and CD20 is expressed in only 20% of cases [10,12,13]. In contrast, HRS cells universally express CD30 and 75–85% of cases express CD15 [14,15,16,17,18,19,20,21]. The reprogramming and loss of the B cell phenotype in HRS cells likely occurs due to genomic alterations that alter important signaling pathways, including NOTCH-1, Janus kinase and Signal Transducer and Activator of Transcription (JAK/STAT), and nuclear factor kappa B (NF-κB) [22,23,24]. Genomic amplification of *REL*, *MAP3K14*, and *BCL3* and mutations in *NFKBIE*, *NFKBIA,* and *TNFAIP3* contribute to constitutive activation of the NF-κB pathway, promoting the survival of HRS cells [23,25,26,27,28,29]. Constitutive JAK/STAT signaling is also a hallmark of cHL and most commonly occurs as a consequence of amplification of *JAK2* and mutations in *STAT6* [30,31,32]. More recently, whole exome sequencing of HRS cells has revealed that ~90% of cHLs harbor mutations affecting the JAK/STAT pathway (including *STAT6*, *SOCS1*, *STAT3*, *STAT5B*, *JAK1*, *JAK2,* and *PTPN1*) [33]. Other genomic alterations also contribute to HRS survival by implicating the nuclear export of proteins and RNAs (*XPO1*), AKT signaling (*GNA13* and *ITPKB*), and evasion of immune surveillance (*CIITA*, *PDL1*, *B2M*, *CD58,* and *TNFRSF14*) [33]. Finally, HRS cells have genomic instability resulting from ongoing chromosomal rearrangements [34] and disruption of 3D telomere-*TRF2* interaction [35].

The HRS cell represents only ~1% of all cells within the tumor environment, the remaining being composed of various immune cells, such as macrophages, eosinophils, neutrophils, mast cells, fibroblasts, and B and T lymphocytes. The predominant fraction of these immune cells belong to the CD4+ T cell family, specifically T helper 2 (Th2) and T regulatory (Treg) cells [36,37,38]. These immune cells are recruited to this inflammatory milieu by the presence of cytokines and chemokines within the microenvironment. In fact, HRS cells can express and secrete CCL5, CCL17, CCL22, and IL-5, all of which can attract CD4+ T cells into the microenvironment [39]. Once the CD4+ T cells rosette the HRS cells, various ligand-receptor interactions occur, including CD40-CD40L, which has been shown to trigger the NF-κB pathway and lead to further production and maintenance of HRS cell colonies [40,41,42,43].

**Figure 1:**
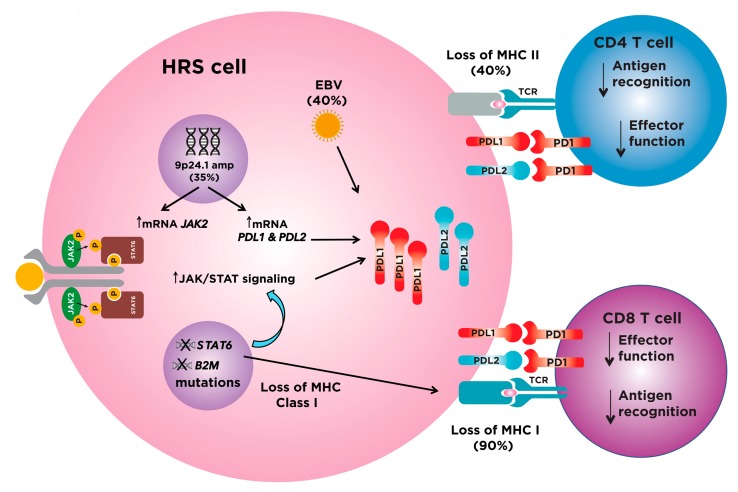
Hodgkin Reed-Sternberg (HRS) cells escape immune detection by over-expressing program death ligands PDL1/PDL2 and silencing Major Histocompatibility Complex (MHC) expression. HRS cells over-express PDL1 and PDL2, both ligands for PD1 on T cells, which once engaged, suppresses T cell effector function. The main mechanisms of over-expression are amplification of 9p24.1, the location of PDL1, PDL2 and Janus kinase 2 (JAK2). Over 90% of classical Hodgkin Lymphomas (cHLs) harbor genetic alterations that may activate JAK/STAT signaling, the most common being JAK2 and STAT6, which can ultimately increase PDL1 expression. Epstein–Bar Virus (EBV) can further increase the expression of PDL1 and PDL2. HRS cells can also promote immune tolerance by silencing the expression of MHC class I and II molecules, which are key to present tumor antigens and activate CD8 and CD4 T cells, respectively. Abbreviations: HRS, Hodgkin Reed–Sternberg; PDL1, program death ligand 1; PDL2, program death ligand 2; PD1, program death 1; MHC I, major histocompatibility complex class I; MHC II, major histocompatibility complex class II; B2M, beta 2 microglobulin, a component of MHC I.

In order to thrive in a lymphocyte-rich microenvironment, HRS cells have developed multiple mechanisms to promote immune tolerance. The most clinically significant mechanism of dampening effector T cell function is by stimulating the programmed death 1 (PD-1)/programmed death ligand 1 (PD-L1) immune checkpoint. PD-L1 expression is variable in cHL patients [44,45], but recent evidence has demonstrated that the level of expression of PD-L1 is associated with the number of copies of the *PDL1* gene locus present on chromosome arm 9p24. High-level amplification, present in a third of HL cases, is associated with the highest expression of PD-L1 protein at the cell surface [46]. The 9p24.1 amplification also contains Janus kinase 2 (*JAK2)* and *PDL2*, which further upregulates PD-L1 expression, the former through activation of JAK/STAT signaling [46]. Finally, Epstein–Barr virus (EBV), present in HRS in 30–40% of classical HL, can also promote the expression of PD-L1 and PD-L2 [47]. More specifically, EBV induces PD-L1 expression through the activation of the transcription factor pathway AP1 and downstream upregulation of c-Jun and JunB, a hallmark pathway of classical HL, irrespective of 9p24.1 copy numbers [48,49]. In addition to hijacking the immune checkpoint system, HRS cells have also evolved other mechanisms to evade immune surveillance. Recent evidence is emerging that highlights a lack of β_2_-microglobulin (B2M) and Major Histocompatibility Complex (MHC) class I at the HRS cell surface in ~90% of cHL cases, which is an essential requirement for an effective CD8+ T cell cytotoxic response [50,51,52]. Furthermore, MHC class II is not expressed on the surface of HRS in ~40% of cHL cases, which is critical for antigen presentation to CD4+ helper T cells [53]. Taken together, HRS cells have adapted multiple mechanisms to evade immune surveillance and thrive in an immune rich milieu (Figure 1).

## 3. Management of Classical Hodgkin Lymphoma

The initial management of cHL is based on the patient’s stage and presence of adverse factors at the time of diagnosis. Positron emission tomography and contrast-enhanced computed tomography (PET/CT) is recommended for initial staging and for subsequent assessment of response to treatment [54]. Chemotherapy remains the standard initial treatment of limited and advanced stage cHL, but the preferred regimen, the number of cycles administered, and the addition of radiation therapy is a matter of debate, which has already been featured in recent reviews [55,56,57]. The two main chemotherapy regimens include either (1) adriamycin (A), bleomycin (B), vinblastine (V), and dacarbazine (D) (ABVD) or (2) bleomycin, etoposide, adriamycin, cyclophosphamide, vincristine, procarbazine, and prednisone (BEACOPP). The two main treatment options for patients with limited stage cHL are either a standard abbreviated course of chemotherapy, typically ABVD, followed by involved field radiation therapy (IFRT) [58,59] or 4–6 cycles of chemotherapy in patients who are deemed not candidates for radiotherapy [60], or a risk-adapted strategy based on the results of an interim PET (iPET) scan performed after 2–3 cycles of ABVD [61,62]. Approximately 80% of patients will be classified as low-risk (iPET-negative), and chemotherapy alone (total 3–4 cycles) without radiation could be considered at a cost of ~5% increased risk of relapse [61]. Combined modality therapy, with possible escalation to BEACOPP, can be considered for the remaining high-risk patients with iPET-positive scans [62]. Patients with advanced stage disease are managed with chemotherapy, either ABVD or BEACOPP, or can follow a risk-adapted approach in which patients are given 2 cycles of ABVD, and, if the iPET is negative, bleomycin can be omitted from the subsequent cycles and if the iPET scan is possible, one can consider escalation to BEACOPP [63,64]. The United States Federal Drug Administration (USFDA) recently approved the addition of brentuximab vedotin (BV) to AVD as an alternative front-line option for patients with advanced HL. BV is an anti-CD30 antibody conjugated monomethyl auristatin E (MMAE) that has significant activity in the relapse setting. In the ECHELON-1 trial, which included 1334 patients with untreated stage III or IV cHL, BV + AVD (AAVD) had a 5% 2 year modified progression-free survival (PFS) advantage over ABVD, but was associated with increased rates of neutropenia and neuropathy [65]. In this trial, 67/1334 (5%) of patients died, with over 90% of events due to treatment-related toxicity, febrile neutropenia in patients treated in the AAVD arm and bleomycin-lung toxicity in the ABVD arm.

The management of patients with relapsed and refractory classical Hodgkin lymphoma (rrHL) that has progressed after frontline therapy is to proceed with salvage chemotherapy followed by autologous stem cell transplant (ASCT) in eligible patients [66]. Multiple salvage chemotherapy regimens have been tested in this setting, but none have been shown to improve overall survival over another [67]. Consolidation with an ASCT in patients with chemo-sensitive disease is considered curative in ~50% of cases [66,68,69,70]. One drug that has been extremely effective in rrHL is BV due to the hallmark feature of increased CD30 expression. The unconjugated anti-CD30 antibody, without MMAE, had minimal clinical activity in patients with rrHL, having an overall response rate (ORR) of 0% [71]. Based on these data, one can conclude that the main clinical benefit of BV is not a consequence of immune-mediated cytotoxicity, but rather the delivery of a potent anti-microtubule inhibitor to HRS cells [71,72]. In patients with rrHL that has relapsed after ASCT, BV has an ORR of 75% and a complete response (CR) rate of 34%. The 5-year PFS and overall survival (OS) was 22% and 41%, respectively [72,73]. Given the impressive response rates of BV in this setting, it has been studied in combination with chemotherapy as part of salvage therapies. For example, BV combined with bendamustine demonstrated impressive results in 2 recent phase I–II trials, with an ORR of 78–93% and CR rate of 43–77% [74,75]. In the study by LaCasce et al., 56% of patients treated with BV and bendamustine experienced infusion-related reactions (IRR) events, which were found to be associated with the production of anti-BV antibodies after cycle 1 in 75% of their patients [75]. BV maintenance can also extend the duration of remission in high-risk patients post ASCT. In the AETHERA phase III trial, maintenance BV extended PFS by 19 months compared to observation alone [76]. There was no overall survival benefit with this strategy and the main toxicities were neutropenia and sensory peripheral neuropathy, which were reversible in most patients.

Taken together, the general approach in treating cHL patients has been to select a treatment that will maximize cure (OS) and minimize long-term toxicity. Unfortunately, these end-points are not often reported in clinical trials due to the extended follow-up time required to capture them. Adding a therapy that has no or very little long-term toxicity to the initial chemotherapy backbone has the potential to improve the outcome of patients with cHL by limiting exposure to subsequent therapy and ASCT that can increase long-term morbidity.

## 4. Immune Checkpoint Inhibitors in Classical Hodgkin Lymphoma

In the past decade, there has been a tremendous success in the field of cancer immunotherapy with the introduction of immune checkpoint inhibitors (ICI). Following exposure to antigen and T cell activation, immune checkpoints (ICs) become expressed at the surface of T cells to inhibit T cell function [77]. This innate mechanism of avoiding damage to self upon chronic antigen exposure is often hijacked by tumors to escape immune detection. The most clinically-relevant IC in cHL is PD-1. PD-L1 expression is not only seen on HRS cells but also on tumor-associated macrophages (TAMs), which may further contribute to so-called T cell exhaustion [44,78]. Engaging PD-1/PD-L1 impairs antitumor T cell function by inhibiting downstream signaling from co-stimulatory receptors and by inducing direct transcription of genes known to suppress T cell function [79]. In addition to PD-1, other ICs are can be expressed at the surface of T cells (e.g., CTLA-4, LAG-3, TIM3, 2B4, CD160, BTLA, and CD112R/TIGIT), further mitigating T cell functions and enforcing T cell exhaustion [79,80,81]. The first ICI to be approved by the USFDA was the anti-CTLA-4 inhibitor ipilimumab for the treatment of melanoma [82]. Upregulation of CTLA-4 on the T-cell competes for the co-stimulatory receptor CD28 thus inhibiting its interaction with B7, thereby inhibiting T-cell activation [83]. In solid tumors, PD-1/PD-L1 engagement occurs in the peripheral tumor micro-environment, whereas the CTLA-4/B7 interaction occurs in the lymph node. This distinction in lymphoma is less clear. In both animal models and pharmacological studies, T cell exhaustion has been shown to be a reversible process with ICI inhibitors [84,85], suggesting it is an effective strategy to restore effector T cell function and cell-mediated cytotoxicity.

Given that cHL is genetically programmed to over-express PD-L1, the first clinical trials were with the PD-1 inhibitors nivolumab and pembrolizumab. Nivolumab was evaluated in a Phase I study (CHECKMATE 039) of 23 patients with rrHL who were heavily pre-treated with systemic therapy, including ASCT in most patients [86]. It had an ORR of 87% and a CR of 17%. Pembrolizumab (KEYNOTE 013) was tested in 31 heavily pre-treated rrHL patients and demonstrated an ORR of 65% with a CR of 16% [87]. Given these encouraging preliminary results, two subsequent phase 2 trials (CHECKMATE 205 and KEYNOTE 087) were designed to further evaluate the clinical efficacy of these PD-1 inhibitors. Both trials evaluated the clinical activity of nivolumab and pembrolizumab in separate cohorts, thus representing the full spectrum of rrHL patients with varying degrees of previous treatment with BV and ASCT [88,89,90]. All cohorts demonstrated a similar response to nivolumab and pembrolizumab, revealing that, regardless of previous treatment history, patients with rrHL retained PD-1 sensitivity and vulnerability. Overall, 70% of patients with rrHL respond to PD-1 inhibitors and 20% have complete responses. The duration of response may be longer with PD-1 inhibitors compared to BV. This hypothesis is being tested in the ongoing Keynote 204 study that randomizes rrHL to receive either BV or pembrolizumab (NCT02684292). In the CHECKMATE 205 study, the median duration of response and PFS to nivolumab was 17 months and 15 months, respectively, which compares favorably to the median PFS of 6 months in patients treated with BV [72,90]. The benefit of nivolumab was not restricted to patients achieving a CR or Partial Response (PR), because even patients with stable disease had a PFS that exceeded 11 months. At a median follow up time of 18 months, the median OS of all patients, including those with “progressive disease”, had not been reached, supporting that PD-1 inhibitors are very beneficial in chemo-refractory HL. As monotherapy, they are also very well-tolerated, with only ~5% of patients experiencing severe adverse events [90]. PD-L1 inhibitors are also active in rrHL. In the phase I JAVELIN Hodgkin trial (NCT02603419), the PD-L1 inhibitor avelumab had an ORR of 55% and CR of 7%, further demonstrating that inhibiting the PD-1/PD-L1 axis is effective in HL [91]. A summary of these trials is presented in Table 1.

Following the impressive results with PD-1 inhibitors as monotherapy, nivolumab was tested in combination with other therapies (Table 1). In the CHECKMATE 039 trial, 31 patients with rrHL received combination nivolumab and ipilimumab. The ORR was 74% and the CR was 19%, which is a similar response achieved with PD-1 blockade alone, suggesting that CTLA-4 inhibition is not the best strategy to improve response rates to PD-1 inhibition. Moreover, patients treated with this combination of ICI experienced more frequent grade 3 immune-related adverse events (irAEs). The combination of nivolumab and BV was conducted in rrHL patients at first relapse, after having failed frontline therapy [94,97]. The ORR of BV and nivolumab was similar to nivolumab alone (82%), but CR rate was significantly higher (61%), and the PFS at 6 months was 91% [94]. Interestingly, 44% of patients had grade 1–2 IRR, which are uncommon with either agent alone. The explanation for these adverse IRRs was unclear, but the authors speculated that given the patients are in the first salvage setting, they may have had an enhanced ability to mount an immune response. BV decreased peripheral CD30+ cells and increased pro-inflammatory cytokines and nivolumab increased the number of circulating T cells, suggesting that BV primes the tumor environment for effective immune infiltration and attack. The safety and efficacy of the combination of nivolumab and AVD in 51 patients with untreated advanced stage cHL or high-risk stage II cHL was recently reported in abstract form [96]. These patients received 4 bi-weekly doses of nivolumab monotherapy followed by nivolumab in combination with AVD. The safety profile of this regimen was similar and consistent with the use of either nivolumab or AVD separately, raising no concerns. With limited follow-up time, the ORR was 84% and the CR rate was 67%. Taken together, PD-1 inhibitors can be safely combined with other therapeutic agents in cHL. Additional work will inform us on the best timing of therapy and the best partner to maximize efficacy without compromising safety.

## 5. Evaluating Response to Immune Checkpoint Inhibitors in Classical Hodgkin Lymphoma

Although the current standard of monitoring disease activity in cHL patients relies on PET/CT [98], limited recommendations exist to guide clinicians in their evaluation of response to ICI. In general, response to therapy has been evaluated using the 2007 [98] and 2014 [99] Revised Response Criteria for Malignant Lymphomas. Although these criteria have been validated in both standard of care and clinical trial populations, they were developed prior to the introduction of ICI therapy. With an increasing number and type of immunotherapy agents under use, an updated criterion labeled as “indeterminate response” was proposed in 2016 to help clinicians in the setting of a common problem encountered with ICIs: “pseudoprogression” [100]. The latter refers to the phenomena of an increased tumor size on imaging or new “lesions”, which might mimic tumor progression by increased tumor bed infiltration by immune cells secondary to immune activation. In the context of lymphoma, this may also occur with immune activation from other causes, such as infections. In this setting, anti-PD-1 therapy can be continued beyond progression of disease (PD) if the patient appears to be deriving clinical benefit, has a good performance status, and has a non-rapid “progression”. Subsequent follow-up imaging or biopsy is required to confirm PD. This approach is deemed safe based on the outcome data collected on 70/243 patients, with rrHL patients treated “beyond progression” on the CHECKMATE 205 trial [90]. The most common reason for reporting PD was the presence of new lesions in 67% of patients. Continued treatment with nivolumab in this setting resulted in the overall reduction in tumor burden in many patients. The median time to next treatment was 17 months and the overall survival for patients treated beyond progression was 84% at 1 year versus 64% in patients who stopped treatment [90]. Thus, improved modalities are clearly needed to evaluate treatment responses in lymphoma patients treated with ICI.

## 6. Biomarkers of Response to Immune Checkpoint Blockade in Classical Hodgkin Lymphoma

In immune-oncology, cHL is the tumor that has the highest response to PD-1 inhibitors. This is likely due to the high expression of PD-L1 due to copy number gains of 9p24.1. A strong intensity of PD-L1 protein expression and amplification of 9p24.1 can be considered as predictive biomarkers of a favorable response to PD-1 inhibitors. This is in contrast to patients with lung cancer, where clinical responses to PD-1 inhibitors can occur irrespective of PD-L1 expression on the tumor cells [101]. Interestingly, amplification of 9p24.1 is also a predictive biomarker of a poor response to conventional chemotherapy. Supporting this finding is that patients with primary refractory cHL in the KEYNOTE-087 study had responses to pembrolizumab that were comparable to the overall rrHL population [102]. In solid tumors, PD-1 inhibitors eliciting a CD8+ mediated cytotoxicity and neoantigen exposure from increased mutation burden is a predictive marker of response [103,104]. However, CD8+ T cells require MHC class I engagement for the cytocidal effect and the latter is lost in >90% of HRS cells. This was confirmed in a recent analysis of biopsies obtained from CHECKMATE 205, where 11/12 responses to nivolumab occurred in patients that lacked MHC class I protein, but expressed MHC class II [105]. Patients with HRS cells harboring amplifications 9p24.1 and express MHC class II on their surface had the highest probability of responding to nivolumab. This study suggests that CD4+ T cells, via an alternative MHC class II-dependent mechanism, play a key role in the response of PD1 blockade therapy.

Other factors, such as elevated leukocyte and eosinophil counts, have been associated with increased risk of progression when treated on PD-1 inhibitors, whereas a lower relative eosinophil count was associated with a reduced risk of progression [106,107].

## 7. Future Directions in the Management of Classical Hodgkin Lymphoma

PD-1 inhibitors are clearly active in rrHL, but there are many unanswered questions regarding the optimal duration and timing of therapy, as well as the best adjuvant therapies that can be safely combined with them to improve efficacy. In patients achieving a CR with PD-1 inhibitors, can therapy be stopped and, if yes, after how many cycles? Two recent studies have reported that a subset of patients who discontinue PD-1 inhibitors while in CR can have long-lasting remissions without relapse beyond a year of follow up [90,108]. If the disease relapses after discontinuing therapy while in CR, is retreatment with PD-1 inhibitors effective? Long-term follow-up of rrHL patients who have remained in CR after discontinuing therapy will provide insight into these issues. Although PD-1 inhibitors are currently approved in cHL patients in the relapse setting, ongoing trials are evaluating them earlier in the course of the disease, e.g., as a pre-transplant salvage regimen or as part of the initial induction therapy. Thus, the best timing to initiate anti-PD-1 therapy and the best combination therapy is an open question.

Treatment regimens that provide good tumor control with limited short and long-term toxicities are needed in HL, especially in older patients that do not tolerate chemotherapy [109,110,111]. Evaluating regimens that include drugs that do not suppress effector immune function seems logical, e.g., drugs that directly target the HRS cell or its micro-environment. Antibody-drug conjugates have the advantage of delivering chemotherapy directly to the tumor cells while decreasing toxicity to immune cells and other tissues. An obvious example of this would be to test BV and a PD-1 inhibitor as a front-line regimen in elderly patients, given that HRS cells appear vulnerable to microtubule inhibition. Radiation has also been safely combined with ICI therapy and may be beneficial in older patients that are less likely to develop the long-term toxicity of radiation. Radiation has been shown to augment response to ICIs, known as the abscopal effect [112]. This phenomenon occurs when targeted radiotherapy to one site leads to disease response in other, non-radiated areas. It has been previously described in other types of cancer [113,114], and is being formally evaluated in cHL (clinical trial NCT03179917). Table 2 summarizes selected ongoing clinical trials testing combination therapy with ICI in cHL (a more comprehensive list can be found in a recent review [115]).

Another area of research is to understand the mechanisms and risk factors that contribute to developing immune-related adverse events (irAEs). Although generally well tolerated, ~5% of cHL patients experience severe, grade 3–4 irAEs to PD-1 inhibitors. There is currently no biomarker or risk factor that can predict which patients are at risk of developing these events. There is also little data on the safety of re-treating patients after an irAE when there is a good anti-tumor response. Patients with auto-immune diseases have been excluded from most ICI trials. However, in the “real world” setting, such patients have been treated with PD-1 inhibitors to treat solid tumors without developing irAEs or “auto-immune flares”.

Given that treatment with PD-1 inhibitors “beyond progression” has benefited many cHL patients, there is an urgent need to improve the modalities used to monitor tumor burden in these patients. An exciting and novel method in cHL currently under investigation is the analysis of circulating tumor DNA (ctDNA). Using deep sequencing technology, ctDNA can be detectable in the plasma of patients with *de novo* cHL and also rrHL [116]. Moving forward, this may be used to risk stratify patients at diagnosis and follow their response in a longitudinal, non-invasive manner. This would also spare patients from recurrent radiation exposure incurred through multiple CT/PET scans. In addition to ctDNA, other biomarkers, such as serum TARC, Galectin-1, and CD163 have been highlighted as potential biomarkers of disease response [29]. These new technological advances may offer clinicians more sensitive tools to monitor cHL tumor burden that transcends the current radiological approach.

## 8. Conclusions

The introduction and refinement of combined chemotherapy and radiotherapy over the last decades have cured most patients with primary cHL. Unfortunately, many of these patients die from treatment-related toxicity, underscoring the need to evaluate effective therapies that have fewer long-term toxicities, which will decrease the rates of secondary neoplasms and cardiac disease. The remarkable clinical activity of PD-1 inhibitors in HL is driven by the genetic reprogramming of HRS cells to evade immune surveillance through the PD-1/PD-L1 axis, with amplification of 9p24.1 and the presence of MCH class II being the most predictive positive biomarkers of response. While PD-1 inhibitors result in a slightly lower CR when compared to BV, the duration of response may be more durable. PD-1 inhibitors are especially effective in patients with primary refractory cHL, suggesting that the immune-mediated cytotoxicity induced by PD-1 blockade is effective in killing chemotherapy-resistant cHL. Given their different mechanism of action and their favorable toxicity profile, PD-1 inhibitors have the potential to increase cures and, importantly, reduce the long-term treatment-related morbidity and mortality in patients with HL.

## Figures and Tables

**Table 1:** Summary of clinical trial data for anti-PD-1 (program death 1) monotherapy, combination immune checkpoint inhibitors (ICI), and ICI + brentuximab vedotin (BV) in cHL patients (classical Hodgkin Lymphoma).

Drug	Phase	ORR%	CR%	Ref.
Monotherapy				
Nivolumab	I	87	17	[86]
Nivolumab	II	68	9	[92]
Pembrolizumab	I	65	16	[87]
Pembrolizumab	II	69	22	[88]
Avelumab	I	54.8	6.5	[91]
Combination Therapy				
Nivolumab + Ipilimumab	I	74	19	[93]
Nivolumab + Brentuximab Vedotin	I/II	82	61	[94]
Ipilimumab + Brentuximab Vedotin	I/II	72	50	[95]
Nivolumab + AVD	II	84	67	[96]

**Table 2:** Summary of all ongoing trials using immune checkpoint inhibitors in combination with other agents in rrHL as of March 1st, 2018. BV = Brentuximab Vedotin; ICE = Ifosfamide, Carboplatin, and Etoposide; ISRT = Involved site radiation therapy.

Combination ICI Clinical Trials	Phase	Trial ID
Nivolumab + Ipilimumab + BV	I	NCT01896999
Nivolumab + Ipilimumab + Daratumumab	I	NCT01592370
Nivolumab + Ipilimumab + Daratumumab + Pomalidomide	I	NCT01592370
Nivolumab + Ibrutinib	II	NCT02940301
Nivolumab + ICE chemotherapy	II	NCT03016871
Pembrolizumab + ISRT	II	NCT03179917
Pembrolizumab + AFM13	I	NCT02665650
Pembrolizumab + Lenalidomide	I/II	NCT02875067
Pembrolizumab + ICE chemotherapy	II	NCT03077828
Pembrolizumab + BV	III	NCT02684292
Pembrolizumab + Vorinostat	I	NCT03150329
Nivolumab + Bendamustine	I/II	NCT03343652
